# “We were here before the Web and hype…”: a brief history of and tribute to the Computational Chemistry List

**DOI:** 10.1186/s13321-018-0322-7

**Published:** 2018-12-18

**Authors:** Frédéric Wieber, Alejandro Pisanty, Alexandre Hocquet

**Affiliations:** 1 Archives Poincaré - Philosophie et Recherches sur les Sciences et les Technologies, UMR 7117 CNRS & Université de Lorraine, Nancy, France; 2 Departamento de Fsica y Quımica Teorica, Facultad de Quımica, Universidad Nacional Autonoma de Mexico, Mexico, CDMX 04510 Mexico

**Keywords:** Computational chemistry, Mailing list, History, CCL, Computational Chemistry List

## Abstract

The Computational Chemistry List is a mailing list, portal, and community which brings together people interested in computational chemistry, mostly practitioners. It was formed in 1991 and continues to exist as a vibrant discussion space, highly valued by its members, and serving both its original and new functions. Its duration has been unusual for online communities. We analyze some of its characteristics, the reasons for its duration, value, and resilience, the ways it embodies and preceded the affordances of online communities recognized elsewhere long after its foundations, and project some aspects into the future. We also highlight its value as a corpus for historians of science.

“Can you imagine that the CCL is more than 10 years old? We were here before the Web and hype…”. This excerpt from a message by Jan Labanowski to the Computational Chemistry mailing List (hereafter referred to as the CCL), written to ask for support to its members, was posted in 2001 [1]. The CCL is now 27 years old and still here now that the hype has died down…

The CCL is a mailing list, portal, and community which brings together people interested in computational chemistry, mostly practitioners. It was formed in 1991 by initiative of Jan Labanowski, at the time a computational chemistry specialist in the Ohio Supercomputing Center, as a mailing list for the hundred persons who had participated in a workshop he had organized together with one of the founding fathers of the field, Charles Bender. The purpose of the list as first created was to continue the lively discussions and encounters that had taken place in the workshop and help grow the field which was accelerating due to the recent availability of maturing quantum, classical and semi-empirical methods, of supercomputers and their power, of personal computers and their flexibility and interoperability, of promising software packages bound to occupy a market niche in the chemical and pharmaceutical industry.

The list has undergone many transformations and survived—even thrived—through them. It went from the original hundred to several thousands members; from a strict, ASCII mailing list to a combination of mailing list, online forum, and portal containing document and software repositories, event announcements, and other communicational resources for the practitioners.

At its core the CCL still is a mailing list, a quaint survivor from early Internet time, in which discussions take place about general principles, practical interpretations of theory, and computational methods. The membership of the list has evolved but continues to hold a mix of theoreticians, computationalists, and experimentalists; of highly experienced practitioners, well known in the field and sometimes founders of it, blended together with young researchers and researchers from communities that are not mainstream for high-powered computational chemistry—young researchers, researchers in industrial laboratories where they may be the only specialist, and researchers in developing countries.

In this respect, the CCL is a typical mailing list of its time as many other flourished in the eighties and nineties in various scientific fields (though a majority of them eventually withered). The genealogy of mailing lists as a communication tool between scientists can be traced back to the times of the fledgling Arpanet. The aim of the computer scientists involved in this project was to develop protocols for the communication between computers. In so doing, they have also built the first tools of human computer-mediated communication. Broadly speaking, the scholarly mailing lists can even be seen as the modern version of the salons of the Enlightment ages, designed by scholars for scholars [2].

Some factors that have made the CCL so durable and resilient through changes are that the communication style is friendly and horizontal; discussions are frank and sometimes sharp but hardly ever hostile; the community is strongly self-regulated for productive discussions; the style and contents imprinted by the founder, Jan Labanowski; the perception of all members that the list provides value; the diversity, broad but not wild, of discussion subjects; and a level of tolerance by members to other members, especially to those less experienced (“n00bs” in Internet parlance) who may inadvertently test established but unwritten rules, restart old discussions or thread well-trodden paths, or become shrill too fast.

The CCL is thus also an exceptional mailing list. Its transparency (the archive is open to all), inclusivity (a poster does not need to subscribe to send a message) and its ethos as designed by a mix of terms of service, moderation practices and moderator personality are key to its persistence. It is particularly important that its definition of topicality allows to blend the theoretical parts of daily practices with the technical parts, and even the commercial ones (something unique to the CCL). The CCL as a tool for the community is thus not only a way to “educate and get educated” (in Labanowski’s words [3]), but also an arena where a vast diversity of topics can be debated.

Pisanty and Labanowski had led a survey of membership from which some of the above conclusions were extracted [4], and Labanowski published about the difficult role of moderator on the CCL [5]. Hocquet and Wieber have more recently discussed the history and activity of the CCL [6]. They have for example explored aspects such as the performative functions of language in the list on the one hand and discourse structure on the other [7].

The language used in Internet fora is neither written nor oral: it has been described as quasi-orality [8]. In scientific mailing lists, it consists in a mix of scholar talk, informal talk and technical talk. In the CCL, this pidgin is perfectly suited to the diversity of topics of concerns to a professionally diverse community. It is notable that most conversations revolve around software as a topic, at the intersection of theories/modelling methods/publications/coding/software support and maintenance/licensing/hardware benchmarking/sales [6].

The “threaded conversation” structure (where the header of a first post defines the topic of a series of answers thus constituting a thread) is a typical and ubiquitous structure of discourse within lists and fora of the Internet. It is pivotal to the structure and topicality of debates within the CCL as an arena. The flame wars (as the liveliest episodes) give valuable and unique information to historians to comprehend what is at stake in the computational chemistry community [9].

Viewed from the present, the CCL has been and continues to be an immensely valuable resource. It also embodied *avant la lettre* some of the affordances that boyd [10], Baym, [11] and many others have identified for social media, such as persistence, replicability, scalability, and searchability. Equally and in consequence the CCL provides amplifying, recording and spreading information and social acts.

Online social media not only comes to mind as an analogy for what the CCL achieves in its combination of mailing list, online forum, and portal, but also as a threat to the CCL in the long term. A number of groups exist on Facebook and LinkedIn, and increasingly also in the online platforms of learned societies like the Institute of Electrical and Electronic Engineers (IEEE) in which discussions on subjects similar to the CCL’s take place. They may oppose less friction to membership, provide richer interactions, and supply software, datasets, literature, and other resources, in formats with which especially younger researchers and students are familiar. However, they have not “caught” enough to displace the CCL, in part because some of the most valued members of the CCL have not migrated to those platforms, either for professional purposes only, or at all. The most valuable resource that keeps the CCL together, in this view, is its own community.

It is a general trend in scholarly communities to give up on mailing lists (as a tool created, designed and maintained by scholars for scholars), like the CCL or the other chemistry related CHMINF-L list [12], and surrender to social media (as services to extract marketing value where scholars are dispossessed of their tools): the CCL is a place of resistance in this respect. There are a few groups and discussions on computational chemistry on Reddit and Facebook which we inspected for this paper but none achieve the function and reach of the CCL; the Reddit discussion thread points to the CCL.

In the future the CCL may face some challenges. Jan Labanowski may retire and leave the stewardship of the community in someone else’s hands. How this would be determined is unclear. A group or forum on an online social media platform may attract enough participants, quality discussions, and resource circulation that members may stop using CCL above the level required to keep it alive (in terms of frequency and of quality of interactions).

Finally, from the historian’s point of view, the issue of the preservation of CCL heritage (and scholar fora heritage in general) is essential. Not only the text of the corpus of messages has to be perennially archived, but also their related metadata, timestamps, headers that define topics, etc. Mailing lists archives are a unique opportunity for historians to explore interactions, debates, even tensions among scientists that reveal a lot about scientific communities: they constitute an important alternative to more official sources such as published papers [13].
